# Desvendando a Ligação entre a Fibrose Hepática Associada ao DHEADM e a Aterosclerose Subclínica

**DOI:** 10.36660/abc.20250503

**Published:** 2025-10-16

**Authors:** Maria Cristina Izar, Francisco Antonio Helfenstein Fonseca

**Affiliations:** 1 Universidade Federal de São Paulo Escola Paulista de Medicina São Paulo SP Brasil Universidade Federal de São Paulo Escola Paulista de Medicina, São Paulo, SP - Brasil; 2 Universidade Federal de São Paulo São Paulo SP Brasil Universidade Federal de São Paulo, São Paulo, SP - Brasil

**Keywords:** Aterosclerose, Fibrose Hepática, Espessura Médio-intimal, Idade Vascular

A doença hepática esteatótica associada à disfunção metabólica (DHEADM) está se tornando rapidamente a doença hepática crônica mais prevalente globalmente, abrangendo o amplo espectro metabólico anteriormente atribuído à doença hepática gordurosa não alcoólica.^1 , 2^ Sua progressão para fibrose e cirrose acarreta não apenas riscos hepáticos, mas também implicações sistêmicas — a principal delas, a doença cardiovascular, agora reconhecida como a principal causa de mortalidade em pacientes com DHEADM.^3 , 4^

O estudo prospectivo intitulado "Relação entre Fibrose Hepática Decorrente da Doença Hepática Gordurosa Associada à Disfunção Metabólica e Aterosclerose Subclínica"^5^ oferece insights oportunos e significativos sobre essa interação hepático-cardiovascular. Ao avaliar marcadores vasculares não invasivos, como a espessura íntima-média da carótida (EIMC) e a idade vascular (IV), os autores fornecem evidências de envelhecimento vascular acelerado em pacientes com DHEADM, particularmente aqueles com fibrose hepática.

Em uma coorte de 114 indivíduos em risco de DHEADM — 84% dos quais eram mulheres, com idade mediana de 64 anos — esteatose foi identificada em 86,8% e fibrose em 27,2%. Pacientes com fibrose apresentaram EIMC e IV significativamente maiores, indicando remodelamento vascular prematuro. Entre os indivíduos com diabetes mellitus tipo 2 (DM2), essa associação foi ainda mais pronunciada: aqueles com fibrose apresentaram EIMC média de 0,742 mm versus 0,653 mm (p < 0,05), e sua IV excedeu a idade cronológica em nove anos.

Esses resultados reforçam descobertas anteriores sobre a carga sinérgica de DHEADM e DM2 em resultados cardiovasculares.^6 , 7^ A inflamação hepática crônica, a resistência à insulina e o estresse oxidativo — todas características da DHEADM — contribuem para a disfunção endotelial e alterações ateroscleróticas.^8 , 9^ Notavelmente, a fibrose hepática representa um estágio da doença sistêmica em que as alterações cardiometabólicas já estão em andamento.

O uso de ferramentas não invasivas, como a elastografia transitória e a ultrassonografia da carótida, como empregadas no presente estudo, oferece valor clínico prático. Essas modalidades contornam as limitações da biópsia hepática, permitindo a avaliação simultânea do risco cardiovascular.^10 , 11^ O artigo de posicionamento de 2019 da Sociedade Brasileira de Cardiologia apoia o uso da EIMC e das placas carotídeas na estratificação de risco, particularmente em indivíduos assintomáticos com comorbidades metabólicas.^12^

Vários estudos, incluindo coortes brasileiras, demonstraram uma forte correlação entre fatores de risco metabólicos — particularmente obesidade visceral e diabetes — e aumento da EIMC, reforçando o uso de imagens vasculares na avaliação de risco para pacientes com DHEADM.^13 , 14^

A IV está ganhando atenção como um substituto mais significativo da saúde arterial em comparação à idade cronológica, particularmente em populações diabéticas e metabolicamente em risco.^15^ Sua elevação no grupo de fibrose DHEADM sugere não apenas dano arterial precoce, mas também a utilidade potencial da IV como um biomarcador dinâmico em estratégias preventivas.

Do ponto de vista clínico, as implicações são claras. Em primeiro lugar, a DHEADM com fibrose deve ser reconhecida como um sinal de alerta para risco cardiovascular elevado. Os hepatologistas devem permanecer vigilantes quanto a complicações vasculares, enquanto os cardiologistas devem incorporar avaliações hepáticas – como escores de fibrose ou elastografia – nas avaliações de risco de rotina, especialmente em pacientes com DM2 ou síndrome metabólica.^4 , 7 , 16^

Em segundo lugar, as intervenções no estilo de vida ganham maior validação. Perda de peso, exercícios e modificações nutricionais continuam sendo a espinha dorsal do tratamento da DHEADM e são conhecidos por influenciar positivamente tanto a histologia hepática quanto a função vascular.^2 , 8^ Agentes farmacológicos em investigação para fibrose hepática também podem oferecer benefícios cardiometabólicos sistêmicos, embora evidências longitudinais ainda estejam pendentes.

Em terceiro lugar, essas descobertas reforçam o apelo por triagem precoce e iniciativas de saúde pública voltadas para a DHEADM em grupos de alto risco. A integração de marcadores de fibrose hepática em protocolos de triagem cardiovascular pode identificar pacientes que, de outra forma, permaneceriam sem diagnóstico até estágios mais avançados da doença.^11 , 14^

Em conclusão, este estudo demonstra que a fibrose hepática associada à DHEADM se correlaciona com aterosclerose subclínica e envelhecimento vascular acelerado. Esses achados destacam a necessidade urgente de abordagens interdisciplinares para lidar com esse crescente impacto na saúde pública. A continuidade da investigação é essencial para determinar se o tratamento da fibrose hepática pode atenuar os desfechos cardiovasculares — uma fronteira em evolução na medicina metabólica.
