# Supplementary material for: Desvendando a Ligação entre a Fibrose Hepática Associada ao DHEADM e a Aterosclerose Subclínica
Source: Arq Bras Cardiol. 2025 Oct 16;122(8):e20250503. [Article in Portuguese] doi: 10.36660/abc.20250503 (PMC12677891; doi:10.36660/abc.20250503)
Supplement: Translation

# Translation: Unveiling the Link between MASLD-Associated Liver Fibrosis and Subclinical Atherosclerosis

**DOI:** 10.36660/abc.20250503i

**Authors:** Maria Cristina Izar, Francisco Antonio Helfenstein Fonseca

**Affiliations:** 1 São Paulo SP Brazil Universidade Federal de São Paulo Escola Paulista de Medicina, São Paulo, SP – Brazil; 2 São Paulo SP Brazil Universidade Federal de São Paulo, São Paulo, SP – Brazil

**Keywords:** Atherosclerosis, Hepatic Fibrosis, Intima-media Thickness, Vascular Age

Metabolically-dysfunction-associated steatotic liver disease (MASLD) is quickly becoming the most prevalent chronic liver condition globally, encompassing the broad metabolic spectrum formerly attributed to non-alcoholic fatty liver disease.^1 , 2^Its progression to fibrosis and cirrhosis carries not only hepatic risks but systemic implications—chief among them, cardiovascular disease, now recognized as the leading cause of mortality in MASLD patients.^3 , 4^

The prospective study titled "Relationship between Liver Fibrosis Due to Metabolic Dysfunction-Associated Steatotic Liver Disease and Subclinical Atherosclerosis"^5^offers timely and significant insights into this hepatic-cardiovascular interplay. By evaluating non-invasive vascular markers such as carotid intima-media thickness (cIMT) and vascular age (VA), the authors provide evidence of accelerated vascular aging in MASLD patients, particularly those with liver fibrosis.

In a cohort of 114 individuals at risk for MASLD—84% of whom were women, with a median age of 64 years—steatosis was identified in 86.8%, and fibrosis in 27.2%. Patients with fibrosis exhibited significantly higher cIMT and VA, indicating premature vascular remodeling. Among individuals with type 2 diabetes mellitus (T2DM), this association was further pronounced: those with fibrosis had a mean cIMT of 0.742 mm versus 0.653 mm (p < 0.05), and their VA exceeded chronological age by nine years.

These results reinforce prior findings on the synergistic burden of MASLD and T2DM on cardiovascular outcomes.^6 , 7^Chronic hepatic inflammation, insulin resistance, and oxidative stress—all hallmarks of MASLD—contribute to endothelial dysfunction and atherosclerotic changes.^8 , 9^Notably, hepatic fibrosis represents a systemic disease stage where cardiometabolic alterations are already in motion.

The use of non-invasive tools such as transient elastography and carotid ultrasound, as employed in the present study, offers practical clinical value. These modalities circumvent the limitations of liver biopsy while allowing simultaneous cardiovascular risk assessment.^10 , 11^The 2019 position paper from the *Sociedade Brasileira de Cardiologia* supports the use of cIMT and carotid plaques in risk stratification, particularly in asymptomatic individuals with metabolic comorbidities.^12^

Multiple studies, including Brazilian cohorts, have shown a strong correlation between metabolic risk factors—particularly visceral obesity and diabetes—and increased cIMT, reinforcing the use of vascular imaging in risk assessment for MASLD patients.^13 , 14^

VA is gaining attention as a more meaningful surrogate of arterial health compared to chronological age, particularly in diabetic and metabolically at-risk populations.^15^Its elevation in the MASLD fibrosis group suggests not only early arterial damage but also the potential utility of VA as a dynamic biomarker in preventive strategies.

From a clinical standpoint, the implications are clear. First, MASLD with fibrosis should be recognized as a red flag for elevated cardiovascular risk. Hepatologists must remain vigilant to vascular complications, while cardiologists should incorporate hepatic assessments—such as fibrosis scores or elastography—into routine risk evaluations, especially in patients with T2DM or metabolic syndrome.^4 , 7 , 16^

Second, lifestyle interventions gain further validation. Weight loss, exercise, and nutritional modifications remain the backbone of MASLD management and are known to influence both liver histology and vascular function positively.^2 , 8^Pharmacologic agents under investigation for hepatic fibrosis may also offer systemic cardiometabolic benefits, though longitudinal evidence remains pending.

Third, these findings bolster the call for early screening and public health initiatives targeting MASLD in high-risk groups. Integrating hepatic fibrosis markers into cardiovascular screening protocols may identify patients who would otherwise remain unrecognized until later stages of disease.^11 , 14^

In conclusion, this study demonstrates that MASLD-associated liver fibrosis correlates with subclinical atherosclerosis and accelerated vascular aging. These findings highlight the urgent need for interdisciplinary approaches to address this expanding public health burden. Continued investigation is essential to determine whether targeting liver fibrosis can attenuate cardiovascular outcomes—an evolving frontier in metabolic medicine.
